# Implementing Integrated Care – Lessons from the Odense Integrated Care Trial

**DOI:** 10.5334/ijic.4164

**Published:** 2018-10-29

**Authors:** Martin Sandberg Buch, Jakob Kjellberg, Christina Holm-Petersen

**Affiliations:** 1 VIVE – Danish Institute for Social Science Research, Copenhagen, DK

**Keywords:** inter-organizational integration, integrated care interventions, implementation, contextual factors, Denmark

## Abstract

**Introduction:**

Creating coordination and concerted action between sectors of modern healthcare is an inherent challenge, and decision makers in search for solutions tend replicate new models across countries and settings. An example of this is the translation of the North West London integrated care pilot into a large-scale trial that took place in the Danish Municipality of Odense from 2013–2016. This article highlights the findings from our evaluation of the ill-fated project and discusses lessons learned.

**Methods:**

We examined implementation and short-term outcome in a multi-method evaluation based on qualitative interviews, direct observation, electronic surveys and quantitative analysis of change in service use and costs, using patient level data and a matched control group.

**Results and discussion:**

Despite an ambitious setup, ample financing, a shared governance structure and a well-functioning project organisation, implementation failed at the clinical level. Also, service use and costs for included patients increased significantly, without yielding the intended results. Primary explanations relate to an overly optimistic timeframe and a failure to take professionals’ wishes, daily practices and values into account. The results underline the importance of basing future attempts at integrated care on thorough studies of the perception of actual needs and timing, including rigorous pilot testing on a smaller scale, before attempting large-scale implementation.

## Introduction

This ‘integrated care case’ is based on the findings of a mixed methods evaluation of a large-scale integrated care model developed and tested in the Municipality of Odense from June 2013 to February 2016 [1]. The evaluation was contracted in August 2014 with the purpose of examining the implementation and short-term results of the project. We highlight the results and performance shortfalls that characterised the project and reflect on lessons learned regarding implementation and dissemination of integrated care models across systems and local settings.

## From North West London to an integrated care model in Odense

As summarized in Table 1, the Odense trial was strongly influenced by the Integrated Care Pilot from North West London 2011–2013 [2,3]^1^ and introduced similar structures for governance and financing, cross-sectorial algorithms for identifying patients at risk, a shared information platform; multidisciplinary patient programmes; multidisciplinary case reviews and a dedicated project management.

**Table 1:** Comparison of main objectives and results of the care model in North West London vs. Odense.

Main elements and objectives	Results	
	North West London	Odense

**Targeted patients**	Patients with type 2 diabetes and elderly patients over 75 years of age.	Work-active patients with stress, anxiety or depression and elderly patients over 70 years of age.
**Establish shared governance structure and align financial incentives**	Involvement of a large number of organisations was achieved. Also, agreements to invest in development and share savings was made. However, the broad scale pilot had a tendency to make-decision making unclear. No savings documented and financial risk primarily carried by hospital sector.	Governance and aligned financial structures was established as intended across involved organisations. Decision-making seemed clear at the organizational level, but translation to middle management and clinical level proved very challenging. Significant increase in costs documented across patient groups as well as organisations.
**Introduce shared care platform that facilitates electronic information sharing**	Roll-out of the integrated care platform was slow, beset by complications and more costly than anticipated.	Roll-out of the integrated care platform was beset by complications and proved more costly than anticipated. Majority of involved professionals find the shared care platform time consuming and associate it with redundant double documentation.
**Risk stratification and shared care plans as a mean to improve and focus care processes**	Professionals support the idea of care planning. However, majority reports dissatisfaction with the extra time required to create plans and only 30% of the total possible plans are made. Efforts to increase number of completed plans also result in seeing the process as a ‘tick box’ exercise’.	General practitioners are sceptical and find stratification tools too imprecise and time consuming to use. As a result, less than 30% of the expected shared care plans are made. Intensive efforts to increase number of completed plans result in plans being made ‘in order to satisfy the project’.
**Multidisciplinary groups as lifting pole for relational coordination and innovation**	Multidisciplinary meetings are time consuming and dominated by general practitioners and consultants. They also tend to focus on individuals and not the configuration of care delivery as a whole. Mechanisms for holding multidisciplinary groups responsible were weak.	In the beginning, meetings are generally viewed as positive. As the project moves on, experienced outcome diminishes. Meetings are time consuming and dominated by doctor dialogue. Focus tends to be on clinical problems related to individuals rather than organizational learning and innovation of collaboration practices.
**Reduce emergency admissions and shift treatment from hospital to primary care.**	No significant changes documented.	No changes in emergency admissions documented. Elderly patients showed a significantly increased use of both ambulatory and stationary hospital services. Both patient groups showed significantly increased use of primary and social services.
**Improve patient experience**	Survey data indicate that patients like the idea of the pilot and that some feel more involved in the decisions about their care. However, response rates were less than 20%, and majority of respondents did not report any change in the delivery of care.	Survey data and interviews document that patients like the idea of the project. The majority of work-active patients feel more involved in the decisions about their treatment and experience a faster and more coherent treatment. Elderly patients generally have an unclear perception of the intervention and few report any change in the delivery of care.
**Improve clinical outcomes**	Some early evidence of improvement in diabetes care and an increase in dementia case finding was documented. No more important health outcomes were identified.	No significant changes documented for elderly patients. Duration of sick leave for work-active patients increased by an average of eight weeks.

On the local level, the project brought together organisations from secondary, primary and community health and social sectors in an effort to increase both horizontal and vertical integration. The targeted patient groups were patients on sick leave due to stress, anxiety or depression and 70+ year-old patients with chronic illnesses. For both groups, the project aimed to improve health outcomes by creating better access to more integrated care outside hospital and by enabling effective cooperation between professionals across organisations. As primary endpoints, the project sought to reduce the duration of sick leave and retain employment for patients diagnosed with stress, anxiety or depression, and to reduce emergency admissions for the elderly chronic patients. Moreover, an overall success criterion was that the care model would produce better results at reduced or similar costs.

As in the London pilot, the Odense project was rooted in three core-principles:

1. A shared and aligned governance structure across sectors that includes contractual obligations to collaborate, in order to integrate and coordinate care across the participating provider organisations.
2. Improved capacity for relational coordination through implementation of a shared care platform, identification of high-risk patients and participation in cross-sectoral, multidisciplinary groups at the local level.
3. Increased patient involvement and proactive patient programmes that focus on creating a fast, individual and coherent treatment and care for participating patients. As a special service, patients could be offered ten consultations with a psychologist free of charge and without a waiting list.

The project involved a university hospital, the community health, social and mental care service providers in the Municipality of Odense and the 116 general practitioners in the Municipality of Odense.

**Figure 1:**
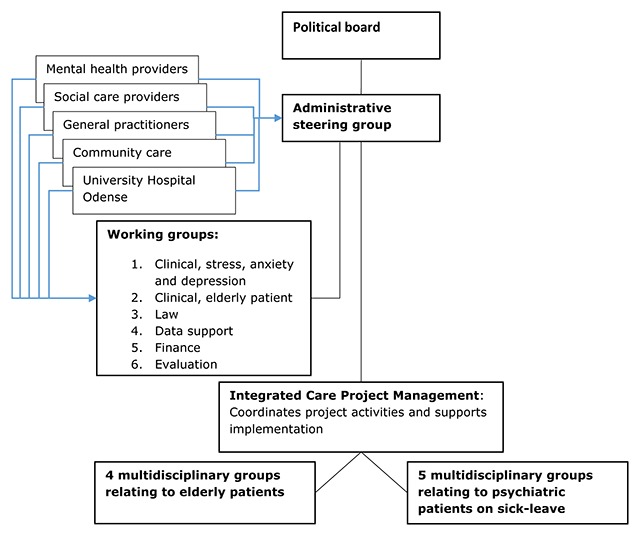
Organisation of the Odense Integrated Care project.

As illustrated in Figure 1, the Odense project was supported by a cross-sectorial governance structure that included a governing board with politicians from the municipality of Odense and the Region of South Denmark, a cross-sectorial steering group, a shared project organisation and a pooled budget of approximately seven million £.

The project was prepared from June 2013 to August 2014, and the trial period ran from August 2014 to February 2016. Participation was mandatory for the community and hospital provider organisations, and costs related to development where covered while costs related to clinical tasks were financed internally. Participation was voluntary for general practitioners, and all their costs related to the project covered. Thirty-three general practitioners covering approximately 56,000 enlisted patients participated in the intervention related to psychiatric patients, while 26 covering approximately 44,000 enlisted patients joined the intervention related to elderly patients. Based on these figures, the general practitioners were expected to represent approximately 1,000 patients on sick leave and 780 elderly medical patients during the 18-month trial period.

### Cross-sectorial and multidisciplinary team conferences

In order to enhance relational coordination, cross-sectoral multidisciplinary teams and meetings were a pivotal point in the project. Nine teams were created, and, as presented in Table 2, each team assembled professionals from all participating parties.

**Table 2:** Participants in multidisciplinary teams.

	Patients on sick leave (5 teams)	Elderly patients with chronic illness (4 teams)

**Private providers**	6–8 general practitioners 2–3 psychologists	6–8 general practitioners
**Municipality and community care**	Community chief physician 2–3 social care workers/job coaches	District nurse Home nurse Dietitian Physiotherapist
**Hospital and specialist care**	Psychiatrist and psychologist with speciality in occupational medicine	Geriatric chief physician Clinical pharmacologist On ad hoc basis: Cardiologist. Endocrinologist. Pulmonary physician.
**Integrated Care project management**	Meeting facilitator	Meeting facilitator

Each team would meet for three hours every other month, and representatives from the supporting project management facilitated the meetings. Each meeting revolved around reviews of four to six particularly complex or illustrative patient cases. The purpose was to create collective learning and to identify ways of improving the quality of the patient path. The teams were mandated to change their own daily practices based on the meetings, but not to change stratification or other project participation requirements. Ideas for change related to project requirements were gathered by the project management and presented to the administrative steering group. Once a year, each team would meet to evaluate team meetings of the preceding year, and once a year all of the teams would meet for a one-day learning seminar.

## Evaluation design

A mixed methods evaluation examined implementation, experienced results and short-term outcomes of the Odense project based on the data sources in Table 3.

**Table 3:** Summary of collected data.

Data collection method	Number completed

Semi-structured group and individual interviews with health care professionals and managers	20 interviews with a total of 77 participants, including 21 GPs
Survey among health care professionals	134 completed in full (77,46% response rate)
Observation of 7 multidisciplinary team meetings	20 hours
Individual interviews with participating patients	19
Survey with patients enrolled in the project	324 completed in full (62,31% response rate)
Patient level data used to analyse service use and costs	Work-active patients: Municipal cost related to sickness benefit, social security and social service. Regional cost and activity related to medicine, general practice and psychiatric care. Elderly patients: Municipal cost related to healthcare, social care and rehabilitation. Regional cost and activity related to medicine, hospital treatment and general practice.

A mixture of observation, interviews and surveys were used to examine involved professionals’ and patients’ perspectives on the project. Interviews were recorded and transcribed in full. To ensure room for new themes to emerge, transcriptions and observation notes were first coded openly by two members of the evaluation team. Subsequently, the identified themes were coded vertically across the collected data and clustered around the initial research questions, presented in **Box 1**:

**Box 1: Research questions**

Interviews and surveys explored the evaluation themes below. Themes were based on the project’s core-principles, dialogue with the project owners and the results from a pilot study conducted by researchers from the University of Southern Denmark [5]:

- The project’s relevance according to participating staff.
- Project organisation, support for implementation and usability of the integrated care platform.
- Multidisciplinary group’s impact on collaboration and capacity for relational coordination.
- Experienced results and suggestions for future improvement.

The coding process did not result in any essential new analytical themes, but subcategories, such as motivation for participation, difficulties in finding relevant patients, barriers related to lack of time and disagreement/uncertainty of inclusion criteria, emerged.

The survey data focused on the same themes as the interviews, and the collected data were analysed using Survey Exact and Microsoft Excel. Ethical approvals were granted by The Danish Data Protection Agency.

The effect evaluation used propensity-score-matching [4] to match included patients to a control group of similar individuals from non-participating general practices from the Municipality of Odense. Matching was based on a broad range of variables related to age, gender, family-status, education, health needs and social service needs prior to inclusion in the project. Short-term impact was measured 12 and 24 months after inclusion, using individual data on each patient’s use of health and social services, end-points related to emergency hospital admissions (for the elderly patients) and duration of sick leave (work-active patients).

## Results

### Pilot phase June 2013-August 2014: Laying the foundation for implementation

In June 2013, to support the many development tasks in the project the partners behind the Odense trial hired a project-team consisting of three consultants and a project director. On the governance-level, agreements concerning information sharing across sectors and contractual agreements regarding economy and patient programmes across participating provider organisations were made. On the operational level, key-professionals and IT providers worked on algorithms for identification of relevant patients, agreeing on and testing criteria for inclusion and the design of a new information-sharing platform.

At this stage, there was a high level of commitment to the project at the managerial level and among the clinicians involved in the working groups. The broad spectrum of health and social workers also supported the vision and principles of integrated care promoted via the project. However, a qualitative pilot evaluation [5] raised serious concerns among participating professionals. These were related to:

- Uncertainty and disagreement regarding stratification tools, inclusion criteria and routines for including patients.
- Widespread concerns regarding the functionality and the risk of double documentation in relation to the shared care platform.

It was clear that these objections constituted potential threats to the planned large-scale implementation. However, the project moved on without the supporting tools and routines being tested in a daily clinical setting. This was partly due to the time constraints that stemmed from the limited span of time in which the partnership had permission to share data across sectors and partly due to a ‘we have to lay the tracks as we move along’ approach in the project organisation. Therefore, the partnership decided on a strategy to improve solutions as part of the ongoing process and to allocate extra resources to implementation support, training and on-location support of the involved professionals.

### The final evaluation

At the end of the trial period, our interviews and surveys showed that a clear majority of the participating professionals positively acknowledged the efforts to support implementation. However, it was also clear that many (pilot) challenges remained unsolved throughout the trial, which increasingly led to uncertainty, lacking motivation and outright resistance among participating professionals. Therefore, it became an important part of our evaluation to seek explanations for the lack of implementation, as presented below.

#### Too few patients included

Inclusion of enough patients and relevant patients turned out to be a major problem throughout the trial, and in the last months inclusion had almost stopped completely. Local activity data – presented in Table 4 – also showed that only 6 of 59 participating general practitioners included the number of patients that were expected as an average. Even more notably, these six practitioners accounted for 40% of all included patients, while the 25 least active general practitioners accounted for only 9%.

**Table 4:** Overview of included and excluded patients compared to initial expectations.

	Included patients	Students that had to be excluded	Patients relevant for data analysis	Expected number of patients	Missing patients	GPs who included more than 20 patients

**Stress, anxiety and depression**	428	174	261	1.000	–739	4
**Elderly patients**	222	–	222	780	–558	2
**Total**	670	174	483	1780	–1297	6

The ensuing lack of patients severely damaged the implementation process in the provider organisations, as the participating professionals met too few patients to become properly acquainted with the project’s care-plan and patient programmes. The lack of patients also led to a great deal of frustration among the other participants, who wondered why the general practitioners – whose participation was voluntary, supported by contractual agreements and ample financing – failed to live up to their responsibilities in the project. A central explanation of these problems was that many general practitioners (and hospital doctors) actively disagreed with the projects inclusion criteria and focus, as explained below.

In relation to patients diagnosed with stress, anxiety and depression, the general practitioners had wished for an intervention that targeted a) an increasing number of students suffering from stress and anxiety and b) socially deprived individuals and families with mental illness. The combination of unmet expectations and inclusion criteria conceived to be imprecise, led to the erroneous inclusion of 174 students in the first months of the trial (as shown in Table 3) and a yet unidentified number of patients with longer and more complicated disease-history. At a later date, the inclusion criteria were clarified and the students excluded. However, this adversely affected many general practitioners’ commitment to the project, as they felt that their professional integrity was compromised, when they were unable to keep their promises to their patients.

In relation to the elderly patients, the broad term ‘elderly patients’ and the proactive focus on relatively well-functioning patients challenged the participating doctors on both a practical and a normative level. On the practical level, the general practitioners found the procedures for stratification, the initial consultations and the resulting shared care plan too time consuming to use:

*Realistically, I have spent three to four hours of extra work on including just one patient. For the patients I have included, the project doesn’t really solve any problems that couldn’t have been solved using our existing routines. In my view, the project has been a waste of time and resources*. [General practitioner]

The general practitioners also reported that the initial integrated care consultation was often difficult to translate into a shared plan addressing a meaningful need for the individual patient, because patients were either too well functioning or too preoccupied by ongoing treatment to benefit from this.

On a more normative level, the proactive focus on a broad group of elderly patients, instead of a specific diagnosis or group of complex patients, challenged both general practitioners and hospital doctors because they had wanted a set-up that targeted collaboration on their most complicated patients coordination wise. However, many had experienced the project as a top-down process, with too little time and room for influence:

*In the working group, we tried to address the issue that the target group of elderly medical patients and the purpose of preventing disease development and hospital admittances was very fluffy. On the one hand, it was very vaguely formulated, and on the other hand it turned out that every time we tried to make it more concrete, for instance by targeting those patients that are most often admitted, we were stopped. Therefore, the working group had a very restricted mandate because a political group had pre-decided the setup*. [Senior hospital doctor]

To many participating doctors this process had undermined their motivation to stay actively committed to the project. Many doctors also expressed doubts about the proposed patient programmes yielding the intended results, which again led to the opinion that it would not make sense to allocate large amounts of resources to new services targeting relatively well-functioning and uncomplicated elderly patients.

Faced with these challenges, the daily management of the integrated care project in various ways tried to accommodate to the experiences and feedback of the general practitioners and other parties in the trial. As an example, general practitioners were allowed to include any elderly person if they saw a need for strengthened corporation, in order to increase the number of included patients. Extra reminders, visits to general practitioners and on-site support were also tried, but, as illustrated below, several of the interviewed general practitioners stated that the one-sided focus on inclusion had adverse effects, as they started to include patients without having a proper goal for doing so:

*The project management keeps asking us to include more patients, so I do. However, the three patients I included yesterday do not have any problems I need help with, and I haven’t added any information or goals to the integrated care platform. My goal was to make the project happy*. [General practitioner]

#### Tools for stratification and the shared care platform

The developed stratification tools and associated risk-lists, which were intended to support inclusion in general practice, were largely abandoned early in the trial because they were considered both imprecise and too time consuming to use. Instead, the majority of general practitioners included patients they meet as part of their regular consultations, because this fitted much more easily into their existing work routines. The drawback of this strategy, however, was that many practitioners’ attention to the project quickly faded, and that the ones who did stay active generally had a low adherence to the given criteria for inclusion.

The majority of professionals also continued to associate the integrated care platform with redundant documentation that they had not asked for. This was partly because it provided a parallel means of communication compared to the existing means of two-way electronic communication across sectors. These perspectives were widespread, and the general perception was that information shared in the integrated-care platform did not differ markedly from the usual information.

#### Multidisciplinary team meetings

Due to a large and continuous effort from the project management, 158 patient cases were identified and reviewed at 47 multidisciplinary meetings. In the first half of the trial period, most participants evaluated the meetings positively, and clear signs of increased capacity for relational coordination were documented. The results were especially pronounced in relation to the traditionally conflict-ridden collaboration between general practitioners and staff from the Community Job centre. However, as the trial period progressed it became increasingly difficult to identify learning cases, and most participants experienced a diminishing outcome, as the same – mainly clinical – problems were discussed again and again:

*Even though we have really racked our brains, we have basically discussed the same issues over and over again. Yes, it’s good that the hospital specialists can inform us of new developments in the field of medication, and I have learned some new things. Again, the set-up is excessively large, considering the outcome*. [General practitioner]

At the end of the trial, there was a relatively clear consensus that the cases brought into review were increasingly irrelevant and were pressured into the meetings in order to satisfy project requirements. Furthermore, participants from the municipality actively questioned the need for their attendance of the meetings, as most of the time they played a marginal role in the ‘doctor dialogue’. The general recommendations from the participants was either to rethink the multidisciplinary meetings in order to create room for new learning, or to discontinue the meetings due to lack of relevance.

#### The patient perspective

As in London [6], almost all patients saw the integrated care project as a positive opportunity and most had a clear understanding of its purpose. Virtually no patients had negative experiences with the integrated care programme, and the vast majority of patients felt confident with the knowledge being shared in the integrated care platform. However, it was also clear that virtually no patients associated their participation with increased involvement.

The results differed in relation to the experienced relevance and perceived results between the two groups of patients.

The elderly patients generally had only a vague idea about the contribution of the shared care model, and few were able to pinpoint specific elements or results of their participation. Some also felt that the shared care plan did not deliver as promised and that nothing had happened after their initial consultation.

In contrast, the patients diagnosed with stress, anxiety and depression generally saw the intervention as highly relevant and helpful. Especially the fast track access to a psychologist free of charge and the sharing of information between these psychologists and the general practitioners was associated with positive results.

#### Short-term outcomes

During the first year after inclusion, both patient groups had a significantly increased use of health and social services compared to the control groups. For the patients with stress, anxiety or depression, the main driver for expenses was an average eight-week increase in the duration of their sick leave, which was the opposite of what was expected. For the elderly patients, the main driver for expenses was an average of 2.5 extra ambulatory visits to a hospital and an average increase in costs related to hospital admissions of approximately 1,800 £, and there was no significant change in the ratio for emergency admissions.

When the costs of delivering the integrated care interventions were added, the economic analysis documented an extra cost totalling at least 4,500 £ per work active patient and at least 3,500 £ per elderly patient during the first year after inclusion, without being able to identify any positive results related to the chosen end points.

#### Termination of the project

Based on the predominantly disappointing results of the evaluation, the parties behind the Odense project agreed to terminate both interventions at the end of the trial period. It was also decided to do a two year follow-up analysis on the outcomes for both patient groups, in order to investigate whether there was a positive outcome of the investment in integrated care programmes in the longer term. The follow-up was published in November 2017 [7] and showed no significant differences related to job market inclusion or emergency admissions. Moreover, the analysis documented that the increased use of health and social services had disappeared in the second year after inclusion. Thus, the result provided support for the integrated care patients being similar to the control group, and validated the stakeholders’ decision to end the project.

## Discussion

Despite many efforts, implementation of the Odense project failed due to a combination of factors that are well known in implementation research and relatively well described in recent literature on dissemination of integrated care models across contexts [8,9,10,11]. The most important being that the:

- Partnership engaged in what proved to be too many and too ambitious development tasks, without having the time necessary to test and prepare a large scale implementation.
- Development and implementation process was perceived as a top-down approach that reduced ownership and motivation among many participating professionals.
- Tasks and responsibilities given to participating professionals (especially the general practitioners who were assigned a crucial role) were out of touch with their daily routines and preferences.
- Due to the decentralized nature of the Danish practice sector, mechanisms for holding general practitioners accountable were weak and left as a responsibility for the local project management.

Therefore, one way of seeing the failed implementation is that the decision makers behind the integrated care project in Odense failed to consider the local context when they introduced an English model into a Danish setting. A particularly important element in this regard, concerns transferring the care model from an English primary care system – organised around clinical commissioning groups and group practices with practice management and clinical support staff – to a Danish system mainly organised around individual practices with 1–2 practitioners, no management and little clinical support staff. The following unsuccessful struggle to implement agreed upon tasks in general practice also illustrates the importance of assessing the organizational capabilities and motivations of involved parties, before engaging in large-scale implementation [11].

In the wisdom of hindsight, it can further be argued that it was unwise to copy the Integrated Care Pilot from North West London before it was properly evaluated, as the English evaluations had in fact identified a number of the same challenges that faced the Odense project [2,8]. Thus, the Odense experience confirms existing knowledge about the challenges related to development and dissemination of concepts for integrated care.

## Conclusion

The Odense case serves as an important reminder of the need to match large-scale interventions addressing collaboration and corporation with the actual organisation and issues at stake for involved professionals. In concurrence with recent literature [12,13], we also recommend that decision makers behind future projects work to support more successful redesign and adaptation of integrated care models across contexts by:

- Recognizing that planning and implementing large-scale interventions takes time and requires time spent on creating a shared understanding of challenges and possible solutions.
- Finding opportunities to learn from others, but at the same time remember assess how or if new or imported interventions work in their local context.
- Basing the process of change on rigorous pilot testing in a small, but realistic setting, before implementing on a larger scale.

The Odense case also points to the importance of understanding and negotiating the sometimes immense political pressure for improvements and quick fixes [14], which allowed (or forced, depending on the perspective) the Odense project to consistently ignore early warning signs and actively communicate the project as a success all the way to the final evaluation. While it was not part of our evaluation to examine these dynamics, future research into the matter is highly relevant. Finally, it seems relevant to point out that lessons learned from failure, though not always rewarding to share, are at least as important for future development as documented success. In this regard, the priority of a rigorous and multidimensional external evaluation, as that pursued in the Odense project, sets an important example to follow in the future.

## Footnotes

Thus the Odense project was influenced by the initial and focused integrated care pilot in North West London that was later transformed into the more broadly orientated Whole Systems Integrated Care programme.
